# The effect of leukocyte- and platelet-rich fibrin on the bone loss and primary stability of implants placed in posterior maxilla: a randomized clinical trial

**DOI:** 10.1186/s40729-023-00487-x

**Published:** 2023-08-09

**Authors:** Meshkat Naeimi Darestani, Hoori Asl Roosta, Seyed Ali Mosaddad, Siamak Yaghoubee

**Affiliations:** 1 Periodontics Department, Dental Faculty, Semnan University of Medical Sciences, Tehran, Iran; 2 Periodontics Department, Dental Faculty, Tehran University of Medical Sciences, Tehran, Iran; 3 Student Research Committee, School of Dentistry, Shiraz University of Medical Sciences, Shiraz, Iran

**Keywords:** Dental implants, Dental implant stability, L-PRF, Platelet derivatives

## Abstract

**Purpose:**

In this study, we investigated the effects of leukocyte- and platelet-rich fibrin (L-PRF) on implant stability and alterations in the marginal bone surrounding posterior maxillary implants.

**Methods:**

This randomized clinical trial was conducted to compare the variable of L-PRF placement around maxillary implants. Resonance frequency analysis (RFA) was used to evaluate the implant stability immediately after surgery and at 1, 2, 4, 6, 8, and 12 weeks after surgery (*t*_0_ to *t*_6,_ respectively). In addition, the amount of marginal bone changes around the implant at t_6_ was compared with the baseline using periapical radiography.

**Results:**

The RFA outcomes were statistically significant within each group (*P* < 0.001, Eta^2^ = 0.322); however, in none of the follow-ups and immediately after the surgery, there was a significant difference between the two groups in terms of the implant stability quotient (ISQ) scores (*P* > 0.05). At t_0_, the test and control groups' respective mean levels of marginal bone loss around the implants were 0.4836 mm and 0.7343 mm, significantly different from the corresponding values at *t*_6_. On the other hand, marginal bone loss around the implant was not significantly different between the two groups in t_0_ and t_6_ (*P* = 0.532).

**Conclusions:**

L-PRF did not improve the RFA outcomes of implants three months after implant placement, and changes in the ISQ values over time were the same in both groups. In addition, L-PRF had no superior effect on the marginal bone loss around the implants.

*Trial registration number:* The research was registered in the Iranian Registry of Clinical Trials on 22 December 2020 (No: IRCT20200624047906N1), available at http://www.irct.ir

## Background

The primary option for rehabilitating partially or completely edentulous oral cavities is oral implantology [1]. The main prerequisite for the effectiveness of implant therapy is the presence of an adequate bone in the treatment area, both quantitatively and qualitatively, for proper osseointegration [2]. Osseointegration, assessed using the bone-to-implant contact (BIC) value under an optical microscope, refers to direct contact between the bone and the implant [3].

In general, the degree of mineralization, mechanical qualities, and remodeling capability all affect the quality of the bone [4], which is classified into four types, with reducing bone density and strength from type I to type IV [5]. It has been reported that implants' success rate and primary stability in bones with lower qualities (type IV) is lower than that of other bone types [6]. Primary implant stability is the biomechanical stability of implants upon placement. It is controlled by various elements, including the quantity and quality of the bone, the macro/micro design of the implant, the surgical method, and the insertion torque. Following new bone development around the implant's surface over time, biological fixation of the implants to the surrounding bone develops, called secondary implant stability. [7]. Multiple techniques have been recommended to enhance the osseointegration process [8]. According to experimental research, adding molecules or growth factors to the implant surface may increase osteoblastic activity and improve the functional integration of implants [9–11]. Beneficial growth factors could be delivered to the surface of implants and neighboring bones using platelet derivatives collected from the patient's blood [12, 13]. These derivatives include factors such as insulin-like growth factor (IGF), platelet-derived growth factor (PDGF), bone morphogenic proteins (BMPs), transforming growth factor-β1 (TGF-β1), TGF-β2, and vascular endothelial growth factor (VEGF), to accelerate the healing process and promote differentiation and migration of mesenchymal cells in the area [14].

Platelet derivatives can be divided into four main categories based on leukocyte concentration and fibrin structure. These include pure platelet-rich plasma (P-PRP), leukocyte and platelet-rich plasma (L-PRP), pure platelet-rich fibrin (P-PRF), and leukocyte and platelet-rich fibrin (L-PRF) [12]. Leukocytes and cytokines are present in the second-generation platelet concentrate known as L-PRF, which also has a robust fibrin matrix [15]. Choukroun et al. developed the L-PRF [16], and unlike platelet-rich plasma (PRP), it is produced without the use of anticoagulants [17]. In addition, its procedure generates more significant output volumes and is quicker, cheaper, and less technique-sensitive. Besides, the L-PRF robust fibrin mesh prevents it from disintegrating quickly after application and allows for the progressive release of growth factors improving angiogenesis and osteoblastic proliferation and differentiation [15, 18–23]. Various L-PRF indications for oral surgical treatments have been postulated in recent years. One of these treatments involves the application of L-PRF to the implant surface to improve implant stability, followed by improvements in BIC and osseointegration [15]. Another concept is to apply L-PRF over an implant to enhance the thickening of the soft tissues, which would increase the stability of the peri-implant tissues and lessen the loss of marginal bone [24].

Few researchers have examined the impact of PRF on implant stability and bone healing [25, 26]. Due to a fibrin network in PRF, which releases growth factors like PDGF1 and its angiogenesis capacity, the subsequent osteoblastic stimulation is higher in PRF than in PRP [27]. Additionally, PRF is more successful *in-vivo* than PRP in promoting osteoblastic proliferation and differentiation, which speeds up bone regeneration and fortifies nearby bone by progressively releasing autologous growth factors [27, 28]. With this background in mind, the purpose of this study was to quantitatively investigate the effect of L-PRF on the primary implant stability and to determine the extent of marginal bone changes over time following implant placement in the posterior maxillary regions.

## Methods

It was decided to conduct a split-mouth randomized clinical experiment with a 1:1 allocation ratio. Patients referred to the Implant Surgery Department of The Tehran University of Medical Sciences served as the sample source. The Ethics Committee of Tehran University of Medical Sciences authorized the current work (138IR.TUMS.DENTISTRY.REC.1399). Additionally, the research was registered in the Iranian Registry of Clinical Trials at http://www.irct.ir (No: IRCT20200624047906N1). All patients were informed about the specifics of the trial, and everyone gave their written consent before participating.

Based on Tabrizi's study [15] and using the following formula to determine the sample size in clinical trial studies and considering the type I error at 0.05 level and type II error at 0.02 level, the mean (SD) in the intervention group as equal to 88.45 (3.36) and that in the control group as equivalent to 76.15 (2.94), the number of samples for each group was calculated to be 13 and a total of 15 people were included in the study for each group to meet the minimum sample requirement during the study period.

$$n=\frac{( {z}_{1-\frac{\alpha }{2}}+{z}_{1-\beta }{)}^{2}({S}_{1}^{2}+{S}_{2}^{2})}{{({\mu }_{1}+{\mu }_{2})}^{2}}$$

Subjects eligible for inclusion in the study were unilateral or bilateral partially edentulous patients with posterior missing spaces and/or free-end arches requiring dental implants in the maxilla's molars or premolars regions. In the case of unilateral edentulism, both test and control implants were placed on one side of the jaw. Even patients who required more than two maxillary posterior implants, unilaterally or bilaterally, were included in the study. Still, only two of their posterior implants were selected for this study. Patients were screened and chosen based on the inclusion and exclusion criteria listed in Table 1.

**Table 1:** Patient selection inclusion and exclusion criteria

Inclusion criteria	Exclusion criteria
18 years old (minimum age)	Patients with untreated, periodontal diseases
Systemic health (ASA I, II)	Diabetes or any other metabolic condition that affects bone metabolism
Adequate oral care	Pregnancy or lactation
Stable occlusion	Immunosuppressive drugs or corticosteroids use
Adequate bone height and width at the surgical site (at least 7 mm width and 11 mm height)	History of radiotherapy or chemotherapy
Adequate mesiodistal and interocclusal spaces in the edentulous area	Prior history of sinus lift or bone augmentation
At least six months must have passed following tooth extraction	Primary stability less than 25 Ncm during fixture placement

### Preparation of L-PRF

**Fig. 1:**
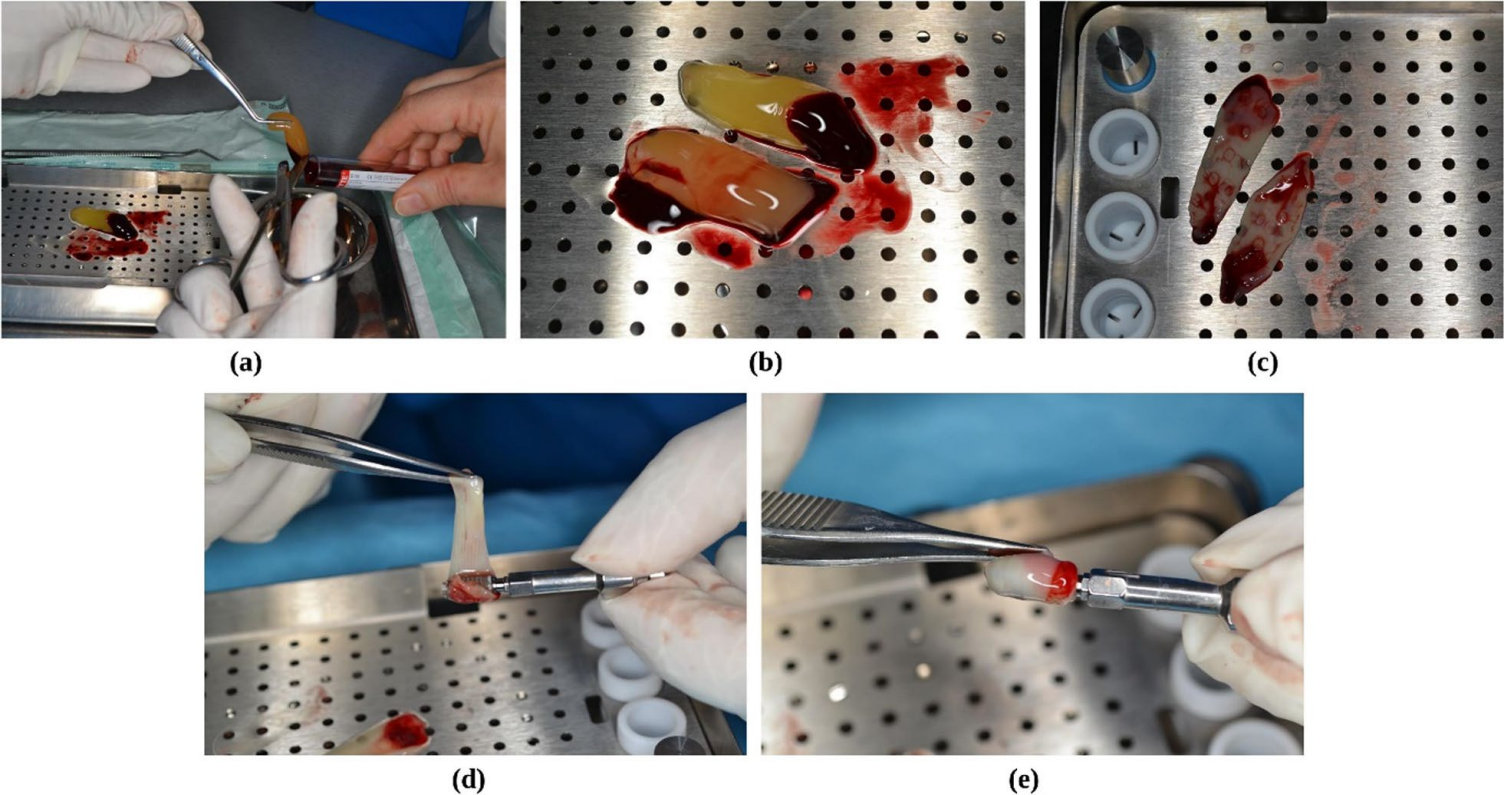
L-PRF preparation. **a** L-PRF removed from the tube, **b** transferred to the PRF box, **c** ready to apply after 5 min, **d**, **e** L-PRF membrane wrapped around dental implants before insertion

Before surgery, 2 × 10 ml of the patient's venous blood was obtained from the antecubital area and centrifuged symmetrically in glass-coated plastic tubes. The tubes were immediately centrifuged with an IntraSpin machine (Intra-Lock International Inc., Boca Raton, FL, USA) for 12 min at 2700 rpm. Forceps were used to remove the fibrin clot that had developed in the center of the L-PRF tubes. Then, the L-PRF was separated from the red blood cell clot discovered right underneath (Fig. 1a), then transferred to the PRF box (Fig. 1b), and the Xpression tray of the Intraspin system was placed on it. After five minutes, the obtained membrane was ready for use at the surgery site (Fig. 1c).

### Surgical method and randomization

All patients underwent surgery by a single surgeon aimed at eliminating operative variables. Local infiltration was injected buccally and palatally at both test and control sites using 2% lidocaine with 1:100,000 epinephrine. Under anesthesia, a crestal incision was made on the desired areas, followed by buccal and palatal full-thickness flap elevation to expose the alveolar crest. Both implantation areas were prepared according to the protocol (25 Ncm, 800 rpm) proposed by the implant manufacturer (Dentium Co., Seoul, Korea).

Random allocation software was used to produce the random sequence. A randomized allocation table was also generated using balanced block randomization. Once the research statistics partner had prepared the randomized allocation list, sealed envelopes numbered sequentially by the practitioner using the letters "T" (indicating the test group) or "C" (meaning the control group) were given to patients before embedding the fixture into the sockets immediately following osteotomy of both implant sockets. Right before placing the implant fixture, the surgeon realized which group (control or test) the surgical site belonged to. Since surgery outcomes were detectable by the patient and the surgeon, there was no possibility of blinding, and only the statistician could be blinded. Control and test sites were chosen entirely randomly using a randomization list.

Next, the L-PRF membrane was wrapped around the fixture (Fig. 1d, e) in the test group and placed at the prepared osteotomy site according to the manufacturer's specifications (Dentium Co., Seoul, Korea). Implants were of similar dimensions (diameter: 4 mm; length: 10 mm) and were inserted in maxillary premolars or molars regions. The implants used in this study were bone-level platform switching Implantium implants (Dentium Co., Seoul, Korea) with a threaded root-form macro design and a sandblasted and acid-etched (SLA) micro design. In the control area, a fixture of the same size was placed without the prior use of L-PRF. Both fixtures were placed 1 mm sub-crestally and then tightened with a torque wrench until both fixtures had reached the desired primary stability (25 Ncm). On both fixtures, a healing abutment was positioned. Using a 4-0 monofilament nylon suture (Supalon, Supa, Iran), the buccal and palatal flaps were re-attached for a passive primary closure. One session was used to operate on each patient. After surgery, all patients were given instructions to use 400 mg of ibuprofen (an NSAID, three times a day for two days), 500 mg of amoxicillin (a systemic antibiotic, three times a day for five days), and 0.2% chlorhexidine mouthwash (twice daily for one week). Two weeks after surgery, all sutures were removed.

### Implant stability measurement

**Fig. 2:**
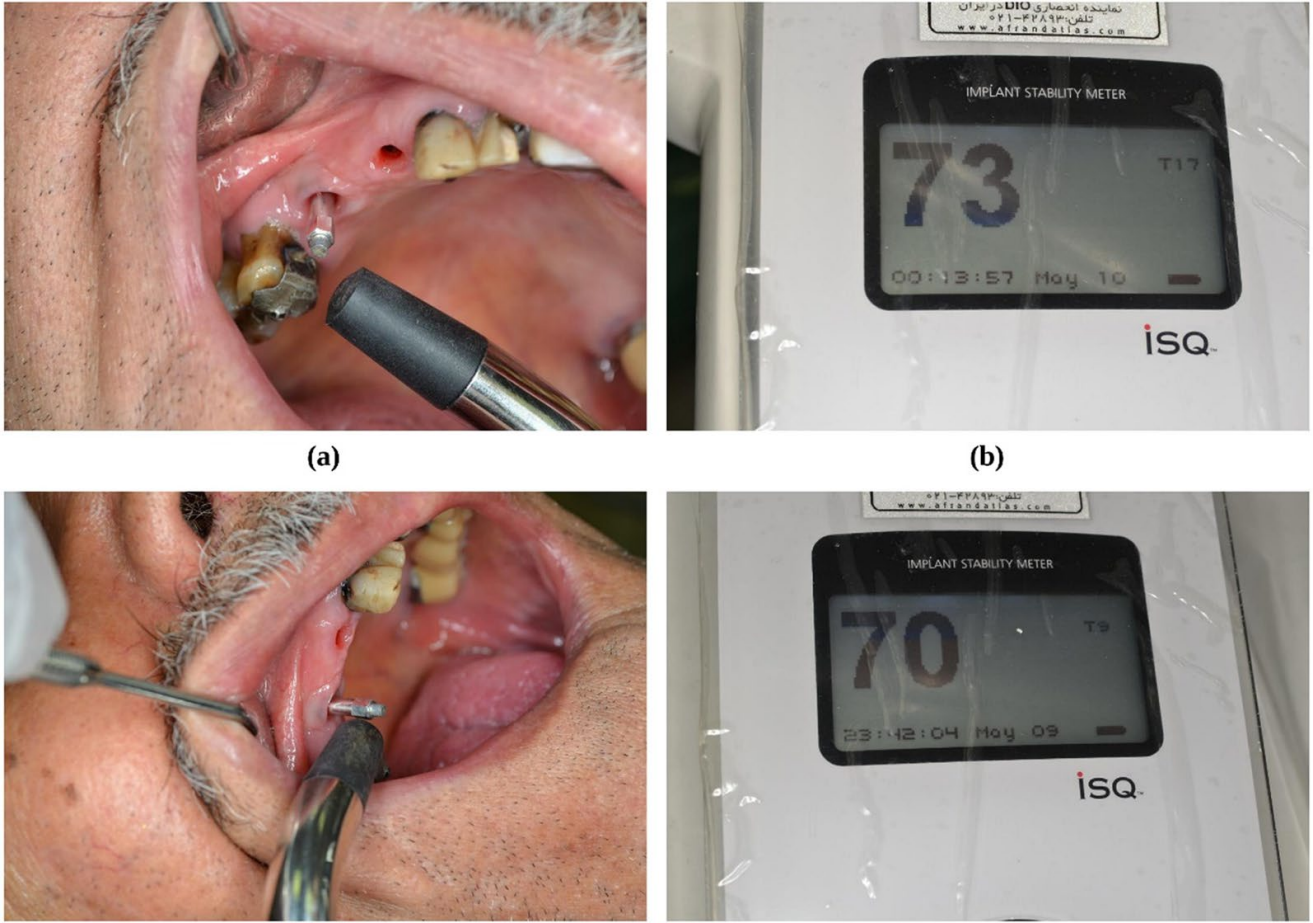
Measuring implant stability using Osstell® device. **a** connecting the SmartPeg converter to implants from different aspects. **b** recording ISQs

**Fig. 3:**
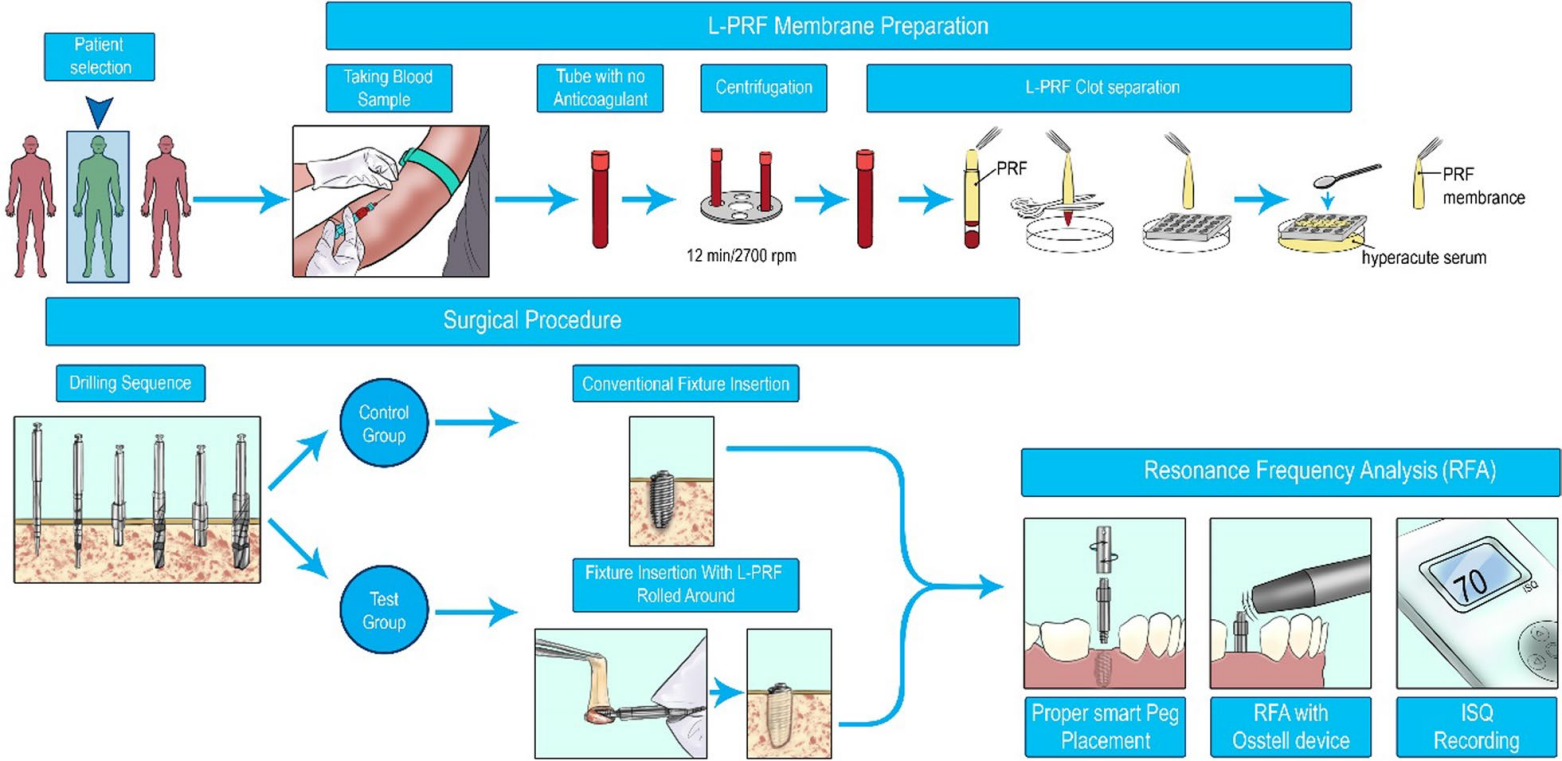
Graphical abstract illustrating the method of performing the study

The resonance frequency analysis (RFA) technique assessed the implant stability. By attaching its transducer (SmartPeg) to the fixture, the Osstell® device (Osstell, Gothenburg, Sweden) was used to take measurements (Fig. 2a, b). The measurements range from 1 to 100, and a higher number on the implant stability quotient (ISQ) scale denotes a more stable implant. Four buccal, palatal, mesial, and distal ISQs were recorded for each fixture, and the mean value was reported for each implant. The RFA measurements were done right away following the surgery (*t*_0_) and repeated one (*t*_1_), two (*t*_2_), four (*t*_3_), six (*t*_4_), eight (*t*_5_), and 12 weeks (*t*_6_) after that. As a graphical abstract, Fig. 3 illustrates the whole procedure performed in this study.

### Radiographic evaluation

**Fig. 4:**
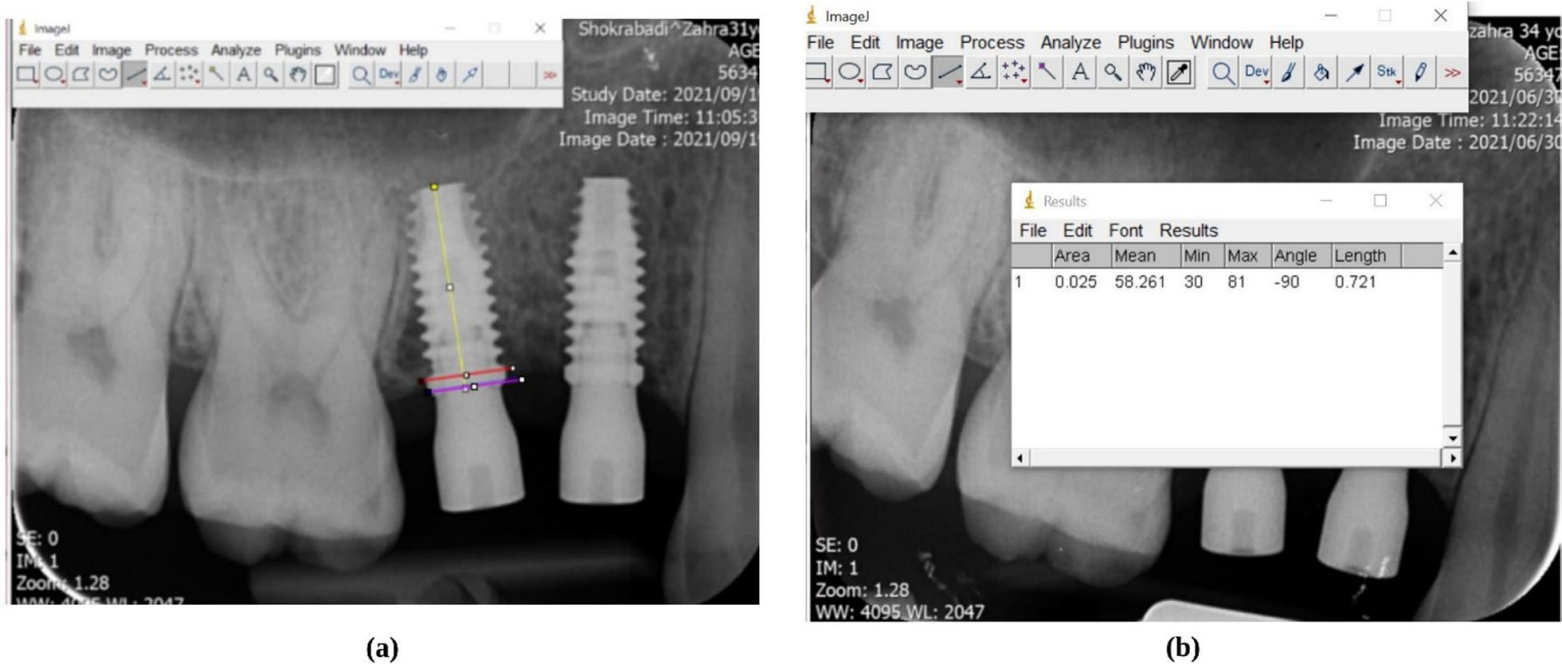
**a** Measurement lines, purple line demonstrating the fixture platform as a fixed reference line; the bone level was defined as the pink line, the point of maximum coronal contact between the bone and the implant. **b** recording data

Immediately after surgery, the placed implants were assessed in all patients employing standardized periapical radiography (KODAK 2100 Intraoral X-Ray System and KODAK RVG 5200 Sensor, Carestream, USA), which was repeated 12 weeks after surgery (t_6_). To standardize periapical radiographic images, both images were acquired using a film holder (Kerr, Kerr Dental, Switzerland) in a parallel manner. To have the patient close the mouth in a similar pattern at both radiography sessions, a high-viscosity additional silicone putty material (Variotime, Kulzer, Germany) was used for bite registration; it was used to ensure the correct position of the film when acquiring both images. The fixture platform was considered a fixed reference line, and the bone level was defined as the point of greatest coronal contact between the bone and the implant. Finally, the distance between these two points was measured separately for the implants’ mesial and distal sites using ImageJ software (ImageJ, National Institutes of Health, Bethesda, MD, USA) (Fig. 4a, b). In baseline radiographs and those obtained after 12 weeks, the average value of mesial and distal crestal bone changes around the implants was measured and compared. To calibrate the measurements in radiographic images, the implant length and diameter, which were already available, were used for calibration.

### Blinding procedure

The patient and surgeon could detect the surgical outcomes, so blinding was not possible. However, during the follow-ups, the assessment clinician who carried out the radiographic examination and the ISQ measurement was unaware of the examined group type (L-PRF or control). Moreover, during data analysis, the statistician who carried out the statistical analyses was unaware of the selective treatment process at each surgical site, and the data analyses were unbiased.

### Statistical analysis

The statistical analyses were done using the SPSS software version 19 (SPSS Inc., Chicago, IL, USA). The ISQ changes in the two groups were measured at various intervals and reported as mean and standard deviation (SD). The ISQ changes were compared within each group and between the two groups using a GLM Univariate with Greenhouse–Geisser (Repeated Measure) method. Furthermore, Analysis of covariance (ANCOVA)(general linear model) was used to compare the marginal bone loss around the implants over time between the two groups and within each group based on the results obtained from periapical radiographs. The statistical significance level was set at less than 0.05.

## Results

**Fig. 5:**
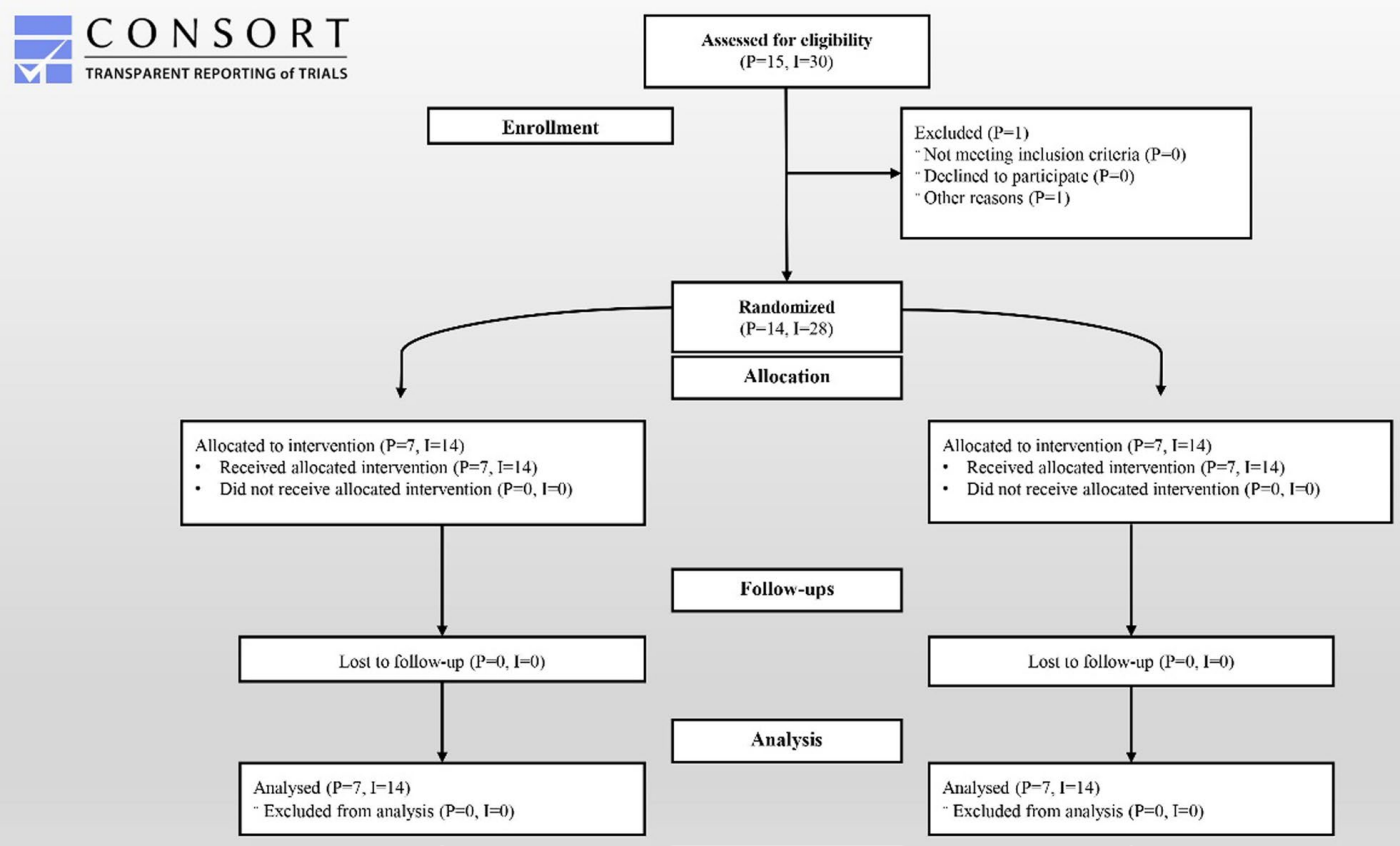
CONSORT flow chart of the studied patients in this investigation. (*P* = Number of Patients, *I* = Number of Dental implants)

This randomized clinical trial study included 15 patients referred to the periodontology department at Tehran University of Medical Sciences with two or more lost teeth in the posterior maxilla region from December 2020 to June 2021. The trial was terminated when the estimated sample size was reached. Following one patient's withdrawal for personal reasons, there were 14 patients with 28 implants, as shown in Fig. 5. Each test and control group consisted of 7 patients with 14 implants. The patients were 35.7% (*n* = 5) males and 64.3% (*n* = 9) females. The average age of patients was 48.93 ± 13.36 years. Table 2 provides an overview of the study participants' demographic characteristics.

**Table 2:** Demographic characteristics of the research participants in the study groups

	Test group	Control group
Number of patients	14	14
Number of implants	14	14
Age (years)	48.93 ± 13.36	
Gender (Male/Female)	M:5/F:9	M:5/F:9
Maxillary region (Premolar/Molar)	P:11/M:3	P:9/M:5
Smoker	None	None

### ISQ changes over time

**Fig. 6:**
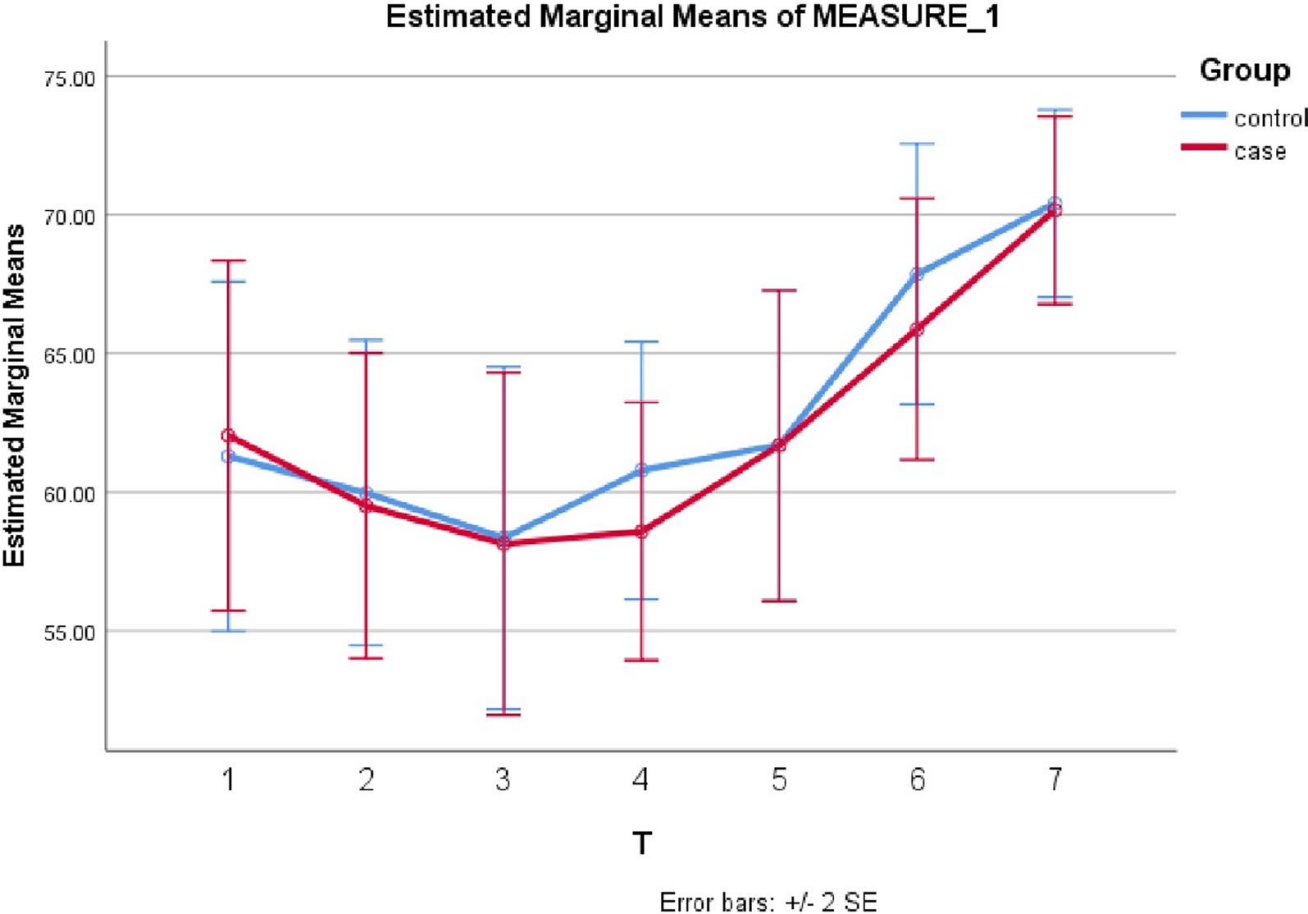
The trend of changes in ISQ values in the studied groups over time

Table 3 depicts the L-PRF and control groups' mean (SD) of ISQ values over time. The mean ISQ values in the test and control groups were 62.0 ± 10.39 and 61.3 ± 13.04 at *t*_0_ (baseline), respectively, and 70.2 ± 7.21 and 70.4 ± 5.29 in the three-month follow-up (*t*_6_). Because Mauchly's sphericity assumption was not established Due to the non-establishment of Mauchly's sphericity assumption (*P* < 0.001), the homogeneity of the variance–covariance matrix was not found, and the Greenhouse–Geisser test results were used. As shown in Fig. 6, the repeated measures test results showed that the intra-group changes in ISQ value were significant over time in both groups (Eta^2^ = 0.322, *P* < 0.001). That is, the intra-group differences in ISQ value between *t*_2_ and t_0_ (*P* = 0.04), *t*_5_ and *t*_0_ (*P* = 0.027), and t_6_ and t_0_ (*P* < 0.001) were statistically significant. Nonetheless, there was no significant inter-group interaction effect (*P* > 0.05). Regarding ISQ value changes over time, the two groups had no significant difference (*P* = 0.833).

**Table 3:** Mean (SD) of ISQ values for implants in the intervention and control groups over time

Group	*T*_0_	*T*_1_	*T*_2_	*T*_3_	*T*_4_	*T*_5_	*T*_6_
	Mean (SD)	Mean (SD)	Mean (SD)	Mean (SD)	Mean (SD)	Mean (SD)	Mean (SD)
L-PRF	62.0 (10.39)	59.5 (8.83)	58.1 (11.30)	58.6 (10.04)	61.7 (10.68)	65.9 (8.89)	70.2 (7.21)
Control	61.3 (13.04)	59.9 (11.56)	58.4 (11.78)	60.8 (7.07)	61.7 (10.25)	67.9 (8.70)	70.4 (5.29)

*T* Test Group, *C* Control Group, *t* time following surgery; *t*_0_ (immediately after surgery, *t*_1_ (one week), *t*_2_ (two weeks), *t*_3_ (four weeks), *t*_4_ (six weeks), *t*_5_ (eight weeks), *t*6 (12 weeks)

### Marginal bone loss

At t_0_, the mean (SD) of marginal bone loss surrounding the implants in the test and control groups was 0.48 (0.39) and 0.73 (0.62), respectively, which was significantly different from the corresponding values reported at t_6_ − 0.28 (0.45) and − 0.30 (0.39) in the test and control groups, respectively (*P* < 0.001) (Table 4). According to descriptive analyses, the mean (standard error) of changes in the studied groups was − 0.340 (0.109) and − 0.240 (0.109), respectively, for the control and the L-PRF groups.

**Table 4:** Comparison of marginal bone loss at t_0_ and t_6_ in the intervention and control groups

Group	Time	Mean	Std. deviation	*t*	*df*	Sig. (2-tailed)
Control	*T*_0_	.7343	.62052	5.900	13	< 0.001
	*T*_6_	−.3004	.39664			
L-PRF	*T*_0_	.4836	.38957	7.815	13	< 0.001
	*T*_6_	− .2796	.45404			

The pairwise comparison of marginal bone loss surrounding the implants in both groups was evaluated using an ANCOVA. After 12 weeks, there were no statistically significant differences in the amount of marginal bone loss surrounding the implants between the intervention and control groups (mean difference = − 0.099; 95% CI: − 0.42, 0.22, *P* = 0.532).

### Intra-observer reliability

Ten samples were measured again in radiographic images to ensure the reproducibility of marginal bone surface measurements around the implants. The intra-class correlation coefficient (ICC) index was calculated for two-way random and absolute-agreement models in SPSS. The calculated ICCs were equal to or greater than 0.995.

## Discussion

L-PRF is one of the most recent new compounds in the category of platelet derivatives [9, 12]. This concentrate contains high levels of growth factors, such as PDGF1, TGFβ1, TGFβ2, VEGF, platelet-derived endothelial growth factors (PD-ECGF), interleukin-1 (IL-1), IL-2, basic fibroblast growth factor (FGF-β), and platelet-activating factor 4 (PAF-4) [14]. Previous studies have investigated the synergistic role of platelet derivatives in bone and soft tissue healing [29–33]. Studies have also examined PRF and its clinical use in several dental fields. PRF treats gingival recessions, periodontal defects, cyst drainage, sinus augmentation, improvement of the width and height of the alveolar bone, ridge preservation, and periodontal defects [9, 34–36]. There are, however, few investigations on the effects of PRF use on the quantity and quality of bone surrounding implants; besides, most of these studies had many intervening factors that might have affected the results.

Since the osteoinductive properties of growth factors such as BMPs and TGF-β in bone healing around the implant have been proven, and PRF is also a rich source of these growth factors, what we expect about the use of this material around the implant is increasing implant stability immediately after surgery, accelerating tissue healing and promoting the formation of new bone at the implant site [37, 38]. Since accurate measurement of these parameters is just possible histologically by tissue samples, it would not be possible to definitively determine the type of healing, time, and process of osseointegration around the implant. But, the clinical manifestation of the healing and ossification around the implant usually appears as the stiffness of the BIC, which can be evaluated using RFA [39]. It can also be presented and compared with ISQ quantitative criteria [40]. In addition, it has been suggested that there is a correlation between ISQ values and histological results [41]. Following site preparation for an implant osteotomy, the primary stability is provided by the implant solely through mechanical interaction with the bone. The mechanical stability of the implant is eventually replaced by secondary or biological stability throughout the healing process. The process of contact osteogenesis and the development of peri-implant woven bone to lamellar bone can explain the increase in ISQ values [42, 43]. Accordingly, the current study aimed to assess L-PRF's impact as a bioactive substance on the bone quality and quantity level around the posterior maxillary implants by measuring clinical stability and marginal bone changes with and without using L-PRF. The intervening and bias-causing factors were adjusted as much as possible.

According to the study findings, the two groups had no significant difference in implant stability. The trend of ISQ changes remained relatively stable over time for the implants in both groups after three months. Additionally, there were no discernible differences between the groups during the three-month follow-up regarding the impact of the L-PRF membrane on marginal bone loss around the implants.

These results contradict the findings of a study by Öncü et al., which investigated the effect of L-PRF on the initial stability of the implant and hard tissue healing. Their results indicated the positive impact of L-PRF on ISQ in one and four-week intervals [25]. The differences between the Öncü study and our study can justify the discrepancy in the data; the implants in that study were all placed in type I and II mandibular bone. Of course, several studies have stated that ISQ values differ for maxillary and mandibular implants [44–47]. Also, the follow-up period in the Öncü study was only one month as it is evident that about one month after the implant placement, the surrounding bone is in the osteoclastic phase, and the implant's stability shows a significant drop compared to the surgical time [48]. A systematic review found that using L-PRF improved implant stability after one week and four weeks, but the difference immediately after insertion was not statistically significant [37].

In the present study, according to the RFA reports, implants in both groups showed descending and ascending trends in the ISQ values over time. The effects of L-PRF on improving stability were insignificant. The L-PRF membrane appears to have been resorbed before causing bone changes around the implant. Since the placement of the L-PRF membrane was done on the fixture when the fixture entered the osteotomized socket, the implant threads first engage with the L-PRF membrane and then with the bone walls, and some of the extra volumes of L-PRF come out of the socket after the complete placement of the fixture. Therefore, the L-PRF membrane remains between the fixture and the bone. Considering that the L-PRF is resorbed about two weeks later [49], it is expected that with the dislodgement of the L-PRF membrane, there will be a micro-gap between the bone and the fixture. During this time, the osteogenic cells have not yet had the opportunity to ossify and fill this micro-gap; on the contrary, the surrounding bone enters the osteoclastic phase, and the implant's stability at this stage is much less than immediately after surgery. Together, these two factors have resulted in the recorded ISQ of the test group implants being lower than that of the control group throughout this time. Finally, after passing this period, we can see that the ISQ values of the implants of both groups have increased over time.

PRP significantly boosted the stability of dental implants within 12 weeks of placement, according to a study by Quesada-Garca et al. [50]. However, there were many intervening factors in the study mentioned above, including patient-related and implant-related variables. In addition, growth factors in plasma were used instead of L-PRF membrane on the surfaces of the implants. On the other hand, research by Ergun et al. and Monov et al. revealed that growth factors high in platelets have no discernible effects on osseointegration [51, 52].

According to the present study, after 12 weeks, the stability of both test and control groups was reported at the same level. This could be due to the limitation of the interfering factors in our study and the split-mouth design for selecting samples. In a study performed by Kapoor et al. [53], between baseline and three months, there was a highly significant rise in ISQ scores in both the PRF and control groups. Our results also agree with this study and another by Diana et al. [54], who also discovered a significant improvement in implant stability over three months in both PRF and control groups. However, there was no significant difference between the groups. A systematic review showed that PRF provided little to no benefit in treating peri-implantitis, implant stability, or guided bone regeneration, as tested in numerous trials [55].

With all these findings, using L-PRF around immediate implants after tooth extraction can be justified; in a previous animal study with a split-mouth design, L-PRF impact on osseointegration around immediate implants in the mandible was compared with a control group [56]. In histological analysis, they confirmed the favorable effect of L-PRF on osteogenesis around implants. It was also reported that in the group which did not have L-PRF to fill the gap between the implant and the extraction socket walls, the apical growth of soft tissue was observed, and the rate of ossification was lower than the L-PRF group. This study suggested that L-PRF could be used as an optimal autogenic source to fill the gap between the extraction socket walls and the implant while improving osseointegration and preventing soft tissue apical growth in the socket; it was also absorbed and removed from the site. Therefore, it did not interfere with the natural process of ossification; the significant difference with our study was the time of implantation and the existing gap between the implant and the bone augmented by L-PRF. The study above justifies the efficiency of using L-PRF in fresh socket implants [56] because the role of L-PRF as a scaffold in the gap is much more vital than its biological properties. In implants placed immediately, the gap between the extraction socket walls and the implant is left alone. Ideally, the blood clot between the implant and the bony wall creates a bone tissue in which the osseous cells may migrate from the socket margin to its center for intramembranous ossification., but after the shrinkage of the clot and its separation from the surface of the fixture, the growth of soft tissue has occurred towards the inside of the cavity disrupting the connection of the clot and bone-forming cells with the implant [57]. As reported, the L-PRF acted as a physical barrier in the area [17, 58], preventing the down growth of soft tissue into the socket. Thus the opportunity for soft tissue intervention in bone repair has been denied. This justification agrees with the results obtained from the study of Benalcázar et al. [59]; the study demonstrated that before implantation, L-PRF placement within wide osteotomies led to increased early bone growth as compared to unfilled wide osteotomies at the early healing time (three weeks in-vivo). In our study, the delayed placement of implants eliminated the need for scaffolding and physical barriers.

In a prospective cohort study conducted by Özveri et al. [60], the use of concentrated growth factor (CGF) around the implant was investigated, and the numerical stability of the implant during surgery and intervals of 1, 2, and 4 weeks was reported by RFA. The study findings showed that CGF does not improve implant stability during the initial healing phase. In an RCT study performed by Gaur et al. [61], three groups (control, PRF, and CGF) were investigated by comparing the stability of immediately placed dental implants using RFA and the bone regeneration around them by measuring radiodensity and the bone gap (horizontal/vertical) on periapical images over time. In agreement with our study, although intergroup results were not significant at any time, they concluded that the treatment of platelet concentrates appeared to improve implant stability. Comparing the quantity (horizontal and vertical gap reduction) and quality (radiodensity/grayscale) of bone regeneration across the three groups showed no statistically significant difference.

In another study by Tabrizi et al. in 2018 on the effect of L-PRF membranes around implants on clinical stability, it was found that the L-PRF membranes could improve the ISQ values of implants during six weeks of follow-up compared to the control group [15]. It should be noted that their follow-up study was shorter than ours, and the results of these studies were even contradictory in six weeks. Among the reasons that may justify the lack of statistically significant difference between the ISQ values of the implants in the test and control groups in our study, we can mention the proximity of the two implants in some patients. Almost in half of the samples of our study, the implants of the test and control groups were located on the same posterior side of the upper jaw, and considering that after the enzymatic breakdown of the L-PRF membrane, fibrinopeptides are used in different ways by the cells in the area around the defect. The breakdown products can apply effective promotional effects locally in the defect and surrounding areas [62, 63]. Therefore, the possibility of unintentionally affecting the implants of the control group from the intervention carried out for the implants of the test group located in the vicinity should also be considered.

In two studies by Kundu et al. [34] and Boora et al. [24] on marginal bone changes around implants associated with L-PRF placement, marginal bone loss around implants was not affected by L-PRF [24, 34]. Moreover, the study by Kundu et al. [34] concluded that implant stability was positively impacted by PRP only at baseline and that there was no significant difference between the two groups in subsequent analyses. In this regard, a systematic review revealed that in the short term, platelet concentrates could considerably increase implant stability and lessen marginal bone loss [64].

Overall, the regional acceleratory phenomenon during drilling and implantation seems sufficient to initiate osteogenesis and induce the growth and differentiation of bone stem cells around implants. Within the limitations of this study, the addition of L-PRF by increasing the dose of growth factors cannot further stimulate osteoprogenitor cells to improve and accelerate ossification. There is probably a physiological range for the induction of cell growth and differentiation by growth and inflammatory factors, which is not limitless or dose-dependent. Therefore, despite all its benefits, L-PRF might not have any additive effects on bone repair around implants. However, large-scale studies with prolonged follow-up periods are still needed to draw firm conclusions.

## Conclusions

In light of the present study's shortcomings, it could be stated that compared to the control group, applying L-PRF did not improve the RFA outcomes in the posterior maxilla three months after implant insertion. Changes in the ISQ values over time were similar in the two groups, and the interaction was not affected by using L-PRF. Besides, the rate of marginal bone loss around the implant was not significantly affected by L-PRF.

## Data Availability

The data supporting this study's findings are available from the corresponding author upon reasonable request.

## Abbreviations

L-PRF: Leukocyte- and platelet-rich fibrin
RFA: Resonance frequency analysis
BIC: Bone-to-implant contact
IGF: Insulin-like growth factor
PDGF: Platelet-derived growth factor
BMPs: Bone morphogenic proteins
VEGF: Vascular endothelial growth factor
P-PRP: Pure platelet-rich plasma
L-PRP: Leukocyte and platelet-rich plasma
P-PRF: Pure platelet-rich fibrin
PRP: Platelet-rich plasma
SLA: Sandblasted and acid-etched
ISQ: Implant stability quotient
SD: Standard deviation
ANCOVA: Analysis of covariance
ICC: Intra-class correlation coefficient
PD-ECGF: Platelet-derived endothelial growth factors
FGF-β: Basic fibroblast growth factor
PAF-4: Platelet-activating factor 4
CGF: Concentrated Growth Factor
